# Machine learning prediction of anastomotic leak after low anterior resection: Nationwide database analysis

**DOI:** 10.1097/MD.0000000000043977

**Published:** 2025-08-22

**Authors:** Takashi Sakamoto, Hideki Endo, Hiroyuki Yamamoto, Takashi Akiyoshi, Ken Shirabe, Hideki Ueno, Hiroshi Hasegawa, Takeshi Naitoh, Yosuke Fukunaga

**Affiliations:** a Department of Gastroenterological Surgery, Cancer Institute Hospital, Japanese Foundation for Cancer Research, Tokyo, Japan; b Department of Healthcare Quality Assessment, Graduate School of Medicine, The University of Tokyo, Tokyo, Japan; c Department of Public Health, Graduate School of Medicine, International University of Health and Welfare, Tokyo, Japan; d The Japanese Society of Gastroenterological Surgery, Tokyo, Japan; e Division of Hepatobiliary and Pancreatic Surgery, Department of General Surgical Science, Graduate School of Medicine, Gunma University, Maebashi, Gunma, Japan; f Database Committee, The Japanese Society of Gastroenterological Surgery, Tokyo, Japan; g Department of Surgery, National Defense Medical College, Tokorozawa, Saitama, Japan; h Project Management Subcommittee, The Japanese Society of Gastroenterological Surgery, Tokyo, Japan; i Division of Gastrointestinal Surgery, Department of Surgery, Kobe University Graduate School of Medicine, Kobe, Hyogo, Japan; j Department of Lower Gastrointestinal Surgery, Kitasato University School of Medicine, Sagamihara, Kanagawa, Japan.

**Keywords:** anastomotic leak, low anterior resection, machine learning, prediction model, rectal cancer

## Abstract

Predicting anastomotic leak preoperatively in patients undergoing low anterior resection for rectal cancer remains a significant challenge. This study aims to develop and validate a predictive model for anastomotic leaks using a machine learning algorithm. Data were collected on patients who underwent low anterior resection from 2015 to 2021 from the National Clinical Database in Japan. The patients were divided into 2 cohorts: a derivation cohort and a validation cohort. The derivation cohort included patients who underwent surgery between January 2015 and December 2019, while the validation cohort included those from January 2020 to December 2021. Three models were developed: logistic regression, logistic regression with least absolute shrinkage and selection operator (Lasso regression), and eXtreme gradient boosting model. We calculated the area under the receiver operating characteristic curve (AUROC) and compared it with the logistic regression model using the DeLong test. A total of 119,818 eligible patients were identified. The incidence of anastomotic leaks was 9.6% in the derivation cohort and 8.4% in the validation cohort, respectively. The predictive ability for the validation cohort using logistic regression (AUROC 0.6324, 95% confidence interval [CI] 0.6220–0.6427, reference) was similar to that of Lasso regression (AUROC 0.6333, 95% CI 0.6229–0.6436, *P* = .13) and eXtreme gradient boosting (AUROC 0.6333, 95% CI 0.6230–0.6437, *P* = .41). Machine learning prediction model for anastomotic leak using preoperative information routinely inputted in the National Clinical Database, showed suboptimal prediction ability. It wound be possible to share the fact with patients that preoperative prediction of anastomotic leak is difficult.

## 1. Introduction

Anastomotic leak remains the most significant complication following low anterior resection. Despite the identification of several risk factors for anastomotic leaks in previous studies,^[1–3]^ preoperative prediction for patients undergoing low anterior resection for rectal cancer continues to be a challenge. Although there have been advancements in technology and surgical techniques, including robotic surgery, the incidence of anastomotic leaks has not decreased.^[4]^ In Japan, the reported incidences of postoperative complications and reoperations following low anterior resection are approximately 10% and 6%, respectively.^[4]^ While diverting ileostomy has been noted to reduce the risk of reoperation due to anastomotic leaks, it carries risks such as dehydration, electrolyte imbalances, and skin issues around the stoma, often necessitating hospital admission. It is crucial to preoperatively identify patients who may benefit from diverting ileostomy based on their risk of anastomotic leaks and to discuss this decision with them.

Several studies have previously developed models to predict anastomotic leaks preoperatively. A nomogram, utilizing data from the Japanese Society for Colon and Rectal Cancer, demonstrated an area under the receiver operating characteristic curve (AUROC) of 0.72.^[5]^ Another study, employing data from the National Clinical Database (NCD) in Japan, achieved an AUROC of 0.64 using a conventional logistic regression model.^[6]^ These outcomes, while informative, were not optimal. A more accurate prediction model is needed for practical application in clinical settings.

Machine learning comprises algorithms that enable computers to learn from data without preestablished models. These methods hold significant potential to enhance prediction and discrimination capabilities beyond empirical analysis. This is achieved through the processing of large datasets and the identification of nonlinear relationships between variables and confounders.^[7]^ Machine learning has been applied in various prediction models within gastrointestinal surgery, particularly for analyzing “big data.”^[8,9]^ NCD is one of the largest repositories, capturing preoperative, intraoperative, and postoperative details, and encompasses over 95% of surgeries conducted in Japan. The potential for a machine learning approach combined with NCD data to achieve optimal prediction capabilities warrants further investigation.

In this study, our objective was to develop machine learning models for predicting anastomotic leaks using NCD data, aiming to enhance predictive ability and compare the performance across different algorithms.

## 2. Methods

We extracted data from the NCD on patients who underwent low anterior resection between 2015 and 2021. The NCD encompasses over 95% of surgeries performed in Japan and has been thoroughly validated in prior research. Further details about the database are discussed in other publications.^[10]^

We excluded patients with the following criteria: concurrent surgeries, age under 18 years, emergency surgeries, low anterior resections not for rectal cancer, and missing data outside of laboratory exams. Concurrent surgeries were classified as operations for malignancies other than rectal cancer, intrapelvic procedures excluding those involving only the ovary, interventions for peritonitis or abscesses, and vascular surgeries (such as embolism treatment, vessel ligation, hemostasis, and stent insertion).

The requirement for informed consent was waived owing to the data’s anonymity. The study received approval from the Institutional Review Board at the Cancer Institute Hospital of the Japanese Foundation for Cancer Research.

### 2.1. Statistical analysis

#### 2.1.1. Splitting dataset into derivation and validation cohort

For the development and validation of our machine learning prediction model, we divided the patients into 2 cohorts: a derivation cohort and a validation cohort. The derivation cohort included patients who underwent surgery from January 2015 to December 2019, while the validation cohort comprised patients from January 2020 to December 2021. We selected clinically relevant candidate predictors from the case report form items, which included patient characteristics (age, sex, body mass index, smoking history, alcohol consumption, and activities of daily living), comorbidities (chronic obstructive pulmonary disease, heart failure, myocardial infarction, angina pectoris, esophageal varices, peripheral arterial disease, hemodialysis, cerebral infarction, and multiple metastatic advanced cancer), history of cardiac interventions (percutaneous coronary intervention and cardiac surgery), preoperative treatments (chemotherapy, radiotherapy, and immunotherapy), preoperative conditions (steroid administration, weight loss > 10%, blood transfusion, sepsis, and American Society of Anesthesiologists physical status), and blood laboratory data (white blood cell count, hemoglobin, platelets, albumin, bilirubin, creatinine, sodium, hemoglobin A1c, and international normalized ratio of prothrombin time [PT-INR]).

#### 2.1.2. Developing prediction models using derivation cohort

The derivation cohort was randomly divided into a training set (70%) and a test set (30%) for the development and internal validation of prediction models. The validation cohort served for the external validation of these models. To predict anastomotic leaks, we developed 3 models: ordinary logistic regression, logistic regression with least absolute shrinkage and selection operator (Lasso regression), and eXtreme gradient boosting (XGBoost).

Lasso regression enhances the standard regression model by allowing for the selection of significant predictors. It does this by reducing the coefficients of less informative variables to zero, thereby focusing on the model’s explainability and ease of interpretation. The regularization parameter, λ (*lambda*), is optimized to maximize the AUROC through 10-fold cross-validation, as implemented in the *glmnet* package.^[11]^ K-fold cross-validation is a technique for assessing model performance by dividing the data into k subsets. In this method, k-1 subsets are used for model development, and the remaining subset is used for validation. This cycle is repeated k times, with the final model performance being the average of the k evaluations.^[12]^

XGBoost employs an ensemble approach, significantly enhancing prediction accuracy by combining multiple decision trees sequentially through gradient descent optimization, facilitated by the xgboost package.^[13]^ An ensemble in machine learning involves the combination of various models to create a superior predictive model. XGBoost is especially effective with structured tabular data. We employed Bayesian optimization to fine-tune performance, carefully selecting the best set of hyperparameters using the *ParBayesianOptimization* package.^[14]^ Bayesian optimization is a refined method for hyperparameter tuning, surpassing traditional approaches like random and grid search by efficiently exploring the hyperparameter space.^[15]^

#### 2.1.3. Measuring prediction performance

To measure the prediction performance, we calculated the AUROC and generated calibration plot for logistic regression, Lasso regression, and XGBoost across both the derivation cohort (including training and test sets) and the validation cohort. Calibration refers to the assessment of agreement between observed and predicted values.^[16]^ Calibration evaluates how well the predicted probabilities match the observed outcomes. A calibration plot visually represents this relationship by displaying the observed (*y*-axis) versus predicted (*x*-axis) probabilities, divided into deciles based on the model’s estimated event probabilities. To compare the performance of the models, we used the DeLong test, with the logistic regression model serving as the reference.^[17]^

An absolute standardized difference of <0.10 indicates a negligible difference between groups.^[18]^ The threshold for statistical significance was established at *P* < .05 for all tests, with all *P* values being two-sided. All analyses were conducted by R version 4.2.2 (2022; R Foundation for Statistical Computing, Vienna, Austria).

## 3. Results

**Figure 1.**
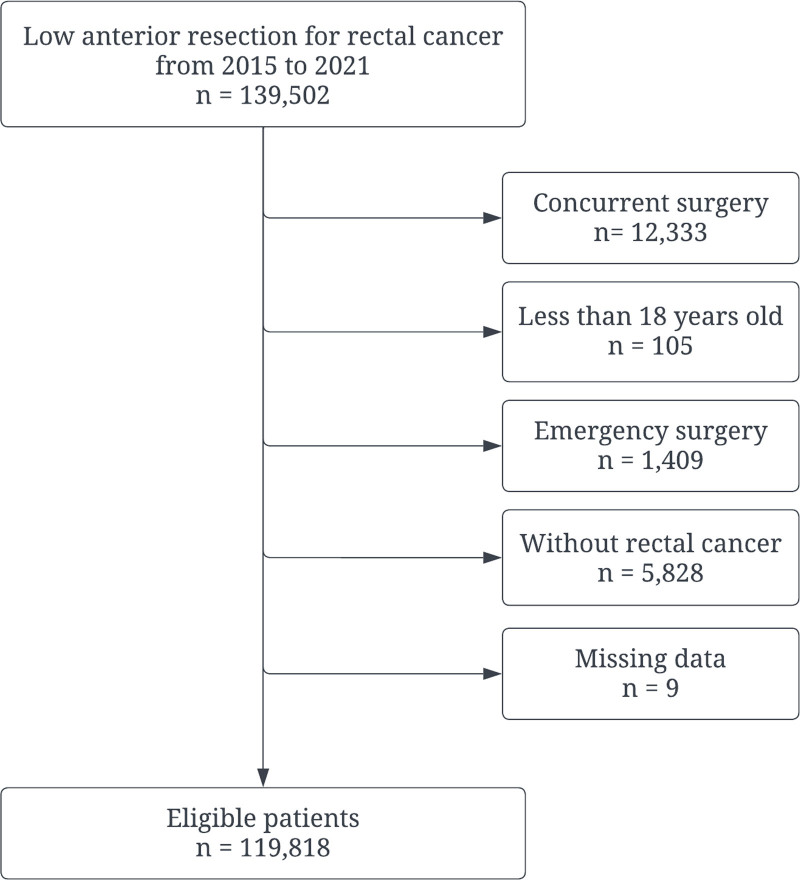
Study flow chart.

We analyzed data from 139,502 patients who underwent low anterior resection between 2015 and 2021. Exclusions were made for concurrent surgery (n = 12,333), age under 18 years (n = 105), emergency surgery (n = 1409), absence of rectal cancer (n = 5828), and missing data (n = 8), resulting in 119,818 eligible patients (Fig. 1). The characteristics of the derivation and validation cohorts are presented in Table 1. Notable imbalances were observed between the cohorts in terms of age categories and hemoglobin A1c levels (absolute standard difference > 0.10). The incidences of anastomotic leak were 9.6% in the derivation cohort and 8.4% in the validation cohort, respectively.

**Table 1:** Patients’ characteristics of derivation and validation cohort.

	Derivation cohort	Validation cohort	ASD
Factors	n = 86,642	n = 33,176	
Patient age, years old			0.12
≤59	18,743 (21.6)	7514 (22.6)	
60–69	28,688 (33.1)	9200 (27.7)	
70–79	27,682 (31.9)	11,687 (35.2)	
80–89	10,848 (12.5)	4430 (13.4)	
≥90	681 (0.8)	345 (1.0)	
Gender			0.016
Male	57,050 (65.8)	21,590 (65.1)	
Female	29,592 (34.2)	11,586 (34.9)	
Chemotherapy within 90 days	6089 (7.0)	2805 (8.5)	0.053
Radiation therapy within 90 days	2542 (2.9)	1222 (3.7)	0.042
Immunotherapy within 90 days	70 (0.1)	47 (0.1)	0.018
Body mass index, kg/m^2^			0.055
18.5<	9880 (11.4)	3740 (11.3)	
≥18.5, 25.0<	56,907 (65.7)	21,115 (63.6)	
25≤, 30<	17,082 (19.7)	7036 (21.2)	
≥30	2773 (3.2)	1285 (3.9)	
Diabetes	16,455 (19.0)	6659 (20.1)	0.027
Smoking within 1 year before surgery	19,688 (22.7)	7727 (23.3)	0.013
Smoking history (Brinkman Index)			0.08
<200	49,861 (57.5)	17,875 (53.9)	
200–1000	27,649 (31.9)	11,748 (35.4)	
1000–1500	6010 (6.9)	2427 (7.3)	
≥1500	3122 (3.6)	1126 (3.4)	
Drinking habit			0.068
Nondrinker (never drinker)	40,706 (47.0)	15,893 (47.9)	
Occasional drinker	18,343 (21.2)	6145 (18.5)	
Habitual	27,593 (31.8)	11,138 (33.6)	
Dyspnea within 30 days before surgery			0.009
No symptom	85,727 (98.9)	32,835 (99.0)	
Shortness of breath during moderate exertion	818 (0.9)	295 (0.9)	
Shortness of breath at even rest	97 (0.1)	46 (0.1)	
ADL within 30days before surgery			0.017
Independent	83,791 (96.7)	32,013 (96.5)	
Partial assistance (including use of equipment, excluding canes etc used daily)	2432 (2.8)	1018 (3.1)	
Full assistance	419 (0.5)	145 (0.4)	
ADL just before surgery			0.012
Independent	83,540 (96.4)	31,924 (96.2)	
Partial assistance (including use of equipment, excluding canes etc used daily)	2639 (3.0)	1079 (3.3)	
Full assistance	463 (0.5)	173 (0.5)	
Chronic obstructive pulmonary disease	3079 (3.6)	1131 (3.4)	0.008
Ascites within 30 days before surgery	800 (0.9)	308 (0.9)	0.001
Esophageal varices within 6 months before surgery	145 (0.2)	57 (0.2)	0.001
Congestive heart failure within 30 days before surgery	464 (0.5)	179 (0.5)	0.001
History of myocardial infarction within 6 months before surgery	313 (0.4)	125 (0.4)	0.003
Angina pectoris within 30 days before surgery	824 (1.0)	244 (0.7)	0.024
History of percutaneous coronary intervention	1957 (2.3)	795 (2.4)	0.009
History of cardiac surgery (excluding pacemaker insertion)	787 (0.9)	345 (1.0)	0.013
History of surgery related to symptoms of peripheral vascular disease	320 (0.4)	117 (0.4)	0.003
Symptoms of peripheral vascular disease	193 (0.2)	73 (0.2)	0.001
Dialysis within 14 days before surgery	455 (0.5)	147 (0.4)	0.012
History of cerebrovascular accident	3178 (3.7)	1535 (4.6)	0.048
Advanced cancer with multiple metastases just before surgery	2089 (2.4)	765 (2.3)	0.007
Long-term steroid administration			0.027
None	85,981 (99.2)	32,859 (99.0)	
Yes (discontinued >30 days before surgery)	80 (0.1)	61 (0.2)	
Yes	581 (0.7)	256 (0.8)	
Weight loss (>10% in the past 6 months)	2045 (2.4)	756 (2.3)	0.005
Bleeding risk factors just before surgery	2751 (3.2)	946 (2.9)	0.019
Preoperative transfusion within 72 hours before surgery	902 (1.0)	347 (1.0)	<0.001
Chemotherapy within 30 days before surgery	1812 (2.1)	842 (2.5)	0.03
Preoperative sepsis just before surgery	74 (0.1)	22 (0.1)	0.007
White blood cell count, µL			0.017
≥9000	5790 (6.7)	2288 (6.9)	
≥3500, <9000	77,992 (90.0)	29,812 (89.9)	
<3500	2685 (3.1)	1030 (3.1)	
Missing	175 (0.2)	46 (0.1)	
Hemoglobin, g/dL			0.025
≥10	78,347 (90.4)	30,128 (90.8)	
7–10	7645 (8.8)	2861 (8.6)	
<7	406 (0.5)	124 (0.4)	
Missing	244 (0.3)	63 (0.2)	
Platelet count, ×1000/µL			0.047
≥35	6537 (7.5)	2900 (8.7)	
18–35	75,700 (87.4)	28,675 (86.4)	
<18	4204 (4.9)	1548 (4.7)	
Missing	201 (0.2)	53 (0.2)	
Albumin, g/dL			0.098
≥4	55,302 (63.8)	20,543 (61.9)	
≥2.5, <4	28,534 (32.9)	11,873 (35.8)	
<2.5	934 (1.1)	389 (1.2)	
Missing	1872 (2.2)	371 (1.1)	
Total bilirubin, mg/dL			0.016
<2.0	81,988 (94.6)	31,404 (94.7)	
≥2.0	4116 (4.8)	1604 (4.8)	
Missing	538 (0.6)	168 (0.5)	
Creatinine, mg/dL			0.041
<1.2	80,931 (93.4)	30,848 (93.0)	
1.2–1.8	3830 (4.4)	1686 (5.1)	
≥1.8	1434 (1.7)	529 (1.6)	
Missing	447 (0.5)	113 (0.3)	
Serum sodium, mEq/L			0.031
≥139	80,208 (92.6)	30,532 (92.0)	
<139	5953 (6.9)	2502 (7.5)	
Missing	481 (0.6)	142 (0.4)	
HbA1c, %			0.199
<6.5	43,994 (50.8)	19,116 (57.6)	
6.5–7.9	8036 (9.3)	3762 (11.3)	
≥8.0	1817 (2.1)	809 (2.4)	
Missing	32,795 (37.9)	9489 (28.6)	
PT-INR			0.024
<1.2	81,328 (93.9)	31,323 (94.4)	
≥1.2	2469 (2.8)	889 (2.7)	
missing	2845 (3.3)	964 (2.9)	
ASA-PS			0.044
1 or 2	77,685 (89.7)	29,292 (88.3)	
≥3	8957 (10.3)	3884 (11.7)	
Anastomotic leak	8278 (9.6)	2799 (8.4)	

ADL = activities of daily living, ASA-PS = American Society of Anesthesiologists Physical Status, ASD = absolute standard deviation, HbA1c = hemoglobin A1c, PT-INR = international normalized ratio of prothrombin time.

**Figure 2.**
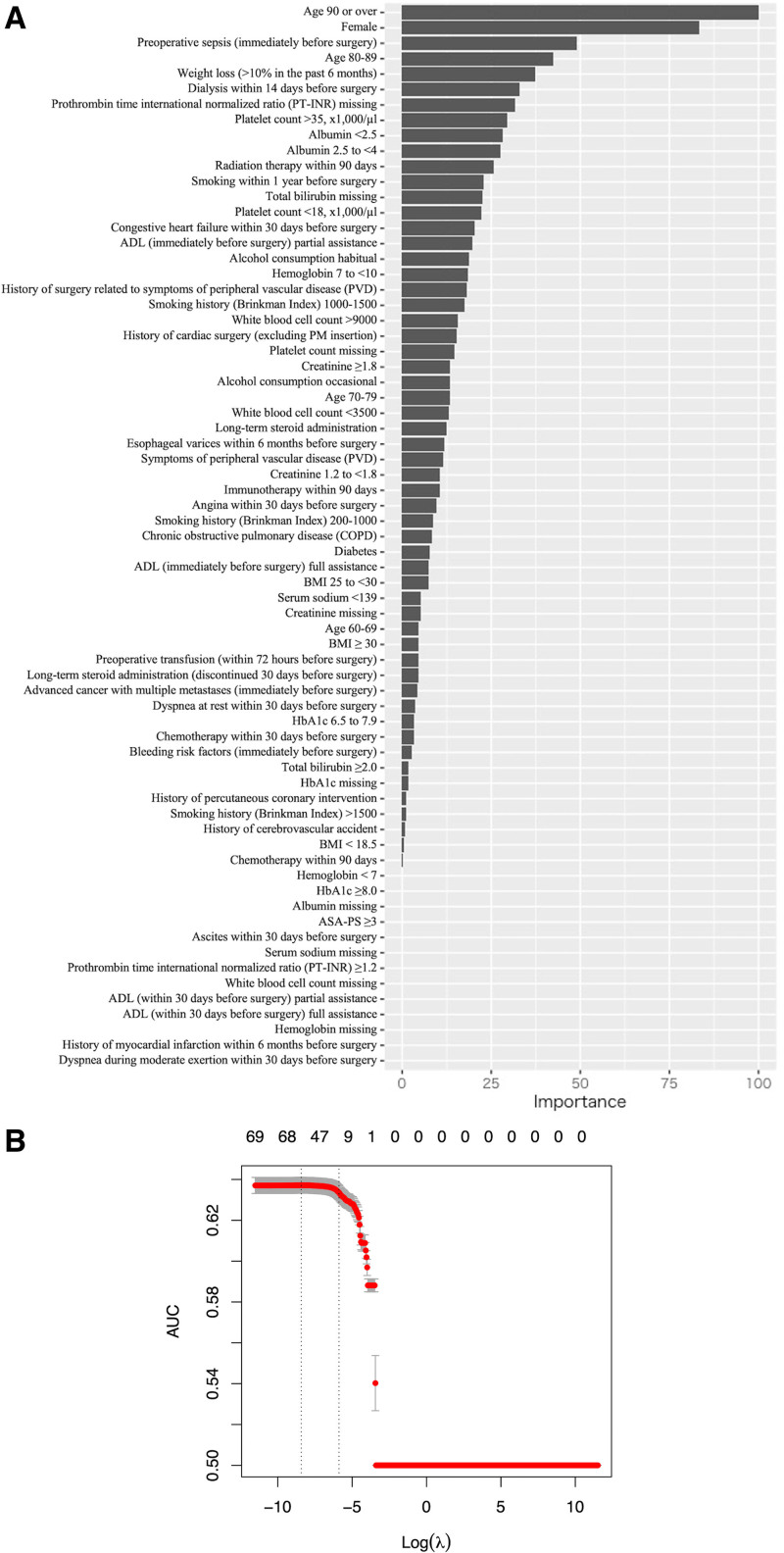
Variable importance (A) and cross validation curve (B) of Lasso regression model. (A, B) The left dashed line indicates the λ that showed the highest AUROC, and the right dashed line represents the largest value of λ for which the error is within 1 standard error of the cross-validated. AUROC = area under the receiver operating characteristic curve.

Table 2 showed predictors and odds ratios identified by Lasso regression. Figure 2A presents the significance of various predictors in Lasso regression, including advanced age, female gender, sepsis, significant weight loss, dialysis, reduced serum albumin levels, and preoperative radiation therapy. Additionally, the absence of PT-INR values and a platelet count exceeding 350 × 10^3^/µL emerged as important predictors.

**Table 2:** Predictors and odds ratio identified by Lasso regression.

Variable	exp(β)	β
Intercept	0.1057	−2.2468
Age 60–69	0.9583	−0.0426
Age 70–79	0.8863	−0.1207
Age 80–89	0.6793	−0.3866
Age 90 or over	0.4029	−0.9090
Female	0.4683	−0.7586
Chemotherapy within 90 days	0.9988	−0.0012
Radiation therapy within 90 days	0.7917	−0.2335
Immunotherapy within 90 days	0.9093	−0.0951
BMI < 18.5	1.0038	0.0038
BMI 25 to < 30	0.9347	−0.0675
BMI ≥ 30	0.9591	−0.0418
Diabetes	1.0714	0.0690
Smoking within 1 year before surgery	1.2308	0.2077
Smoking history (Brinkman Index) 200–1000	1.0828	0.0796
Smoking history (Brinkman Index) 1000–1500	1.1714	0.1582
Smoking history (Brinkman Index) > 1500	0.9890	−0.0111
Alcohol consumption occasional	1.1286	0.1210
Alcohol consumption habitual	1.1849	0.1697
Dyspnea at rest within 30 days before surgery	0.9680	−0.0325
ADL (immediately before surgery) partial assistance	1.1975	0.1802
ADL (immediately before surgery) full assistance	1.0706	0.0682
Chronic obstructive pulmonary disease	0.9265	−0.0763
Esophageal varices within 6 months before surgery	0.8990	−0.1064
Congestive heart failure within 30 days before surgery	1.2027	0.1846
Angina within 30 days before surgery	0.9171	−0.0865
History of percutaneous coronary intervention	1.0116	0.0115
History of cardiac surgery (excluding PM insertion)	0.8693	−0.1401
History of surgery related to symptoms of peripheral vascular disease	1.1784	0.1641
Symptoms of peripheral vascular disease	0.9016	−0.1036
Dialysis within 14 days before surgery	1.3495	0.2997
History of cerebrovascular accident	1.0074	0.0074
Advanced cancer with multiple metastases (immediately before surgery)	1.0405	0.0397
Long-term steroid administration (discontinued 30 days before surgery)	0.9605	−0.0403
Long-term steroid administration	1.1195	0.1129
Weight loss (>10% in the past 6 months)	1.4054	0.3403
Bleeding risk factors (immediately before surgery)	1.0255	0.0252
Preoperative transfusion (within 72 hours before surgery)	1.0414	0.0406
Chemotherapy within 30 days before surgery	1.0296	0.0292
Preoperative sepsis (immediately before surgery)	1.5630	0.4466
White blood cell count > 9000	1.1532	0.1425
White blood cell count < 3500	0.8874	−0.1195
Hemoglobin 7 to < 10	1.1814	0.1667
Platelet count > 35, ×1000/µl	1.3055	0.2666
Platelet count < 18, ×1000/µl	0.8171	−0.2020
Platelet count missing	1.1439	0.1345
Albumin 2.5 to < 4	1.2849	0.2507
Albumin < 2.5	1.2923	0.2564
Total bilirubin ≥ 2.0	0.9830	−0.0172
Total bilirubin missing	0.8148	−0.2048
Creatinine 1.2 to < 1.8	0.9090	−0.0954
Creatinine ≥ 1.8	0.8854	−0.1217
Creatinine missing	0.9548	−0.0463
Serum sodium < 139	0.9539	−0.0472
HbA1c 6.5 to 7.9	0.9692	−0.0313
HbA1c missing	1.0154	0.0153
PT-INR missing	0.7492	−0.2887

ADL = activities of daily living, BMI = body mass index, HbA1c = hemoglobin A1c, Lasso = least absolute shrinkage and selection operator, PM = pace-maker, PT-INR = international normalized ratio of prothrombin time.

Table 3 presents the prediction capabilities of the training, test, and validation cohorts as measured by AUROC. Within the training cohort (70% of the derivation cohort), the AUROC scores were as follows: logistic regression achieved 0.6435 (95% confidence interval [CI] 0.6364–0.6507), Lasso regression scored 0.6425 (95% CI 0.6353–0.6496), and XGBoost registered 0.6419 (95% CI 0.6347–0.6490). In the test cohort (30% of the derivation cohort), the performances were: logistic regression at 0.6288 (95% CI 0.6178–0.6399), Lasso regression at 0.6289 (95% CI 0.6179–0.6399), and XGBoost at 0.6291 (95% CI 0.6180–0.6401).

**Table 3:** Prediction performance of logistic regression and machine learning models.

Model	Derivation (2015–2019, 7:3 split)		Validation (2020–2021)	*P* value*
	Training cohort	Test cohort	Validation cohort	
	n = 60,649	n = 25,993	n = 33,176	
Logistic regression	0.6435 (0.6364–0.6507)	0.6288 (0.6178–0.6399)	0.6324 (0.6220–0.6427)	*Reference*
Lasso regression	0.6425 (0.6353–0.6496)	0.6289 (0.6179–0.6399)	0.6333 (0.6229–0.6436)	.13
XGBoost	0.6419 (0.6347–0.6490)	0.6291 (0.6180–0.6401)	0.6333 (0.6230–0.6437)	.41

Lasso = least absolute shrinkage and selection operator, XGBoost = eXtreme gradient boosting.

* DeLong test.

The predictive performance for the validation cohort, as measured by AUROC, showed that logistic regression (0.6324, 95% CI 0.6220–0.6427, reference) was comparable to both Lasso regression (0.6333, 95% CI 0.6229–0.6436, *P* = .13) and XGBoost (0.6333, 95% CI 0.6230–0.6437, *P* = .41).

**Figure 3.**
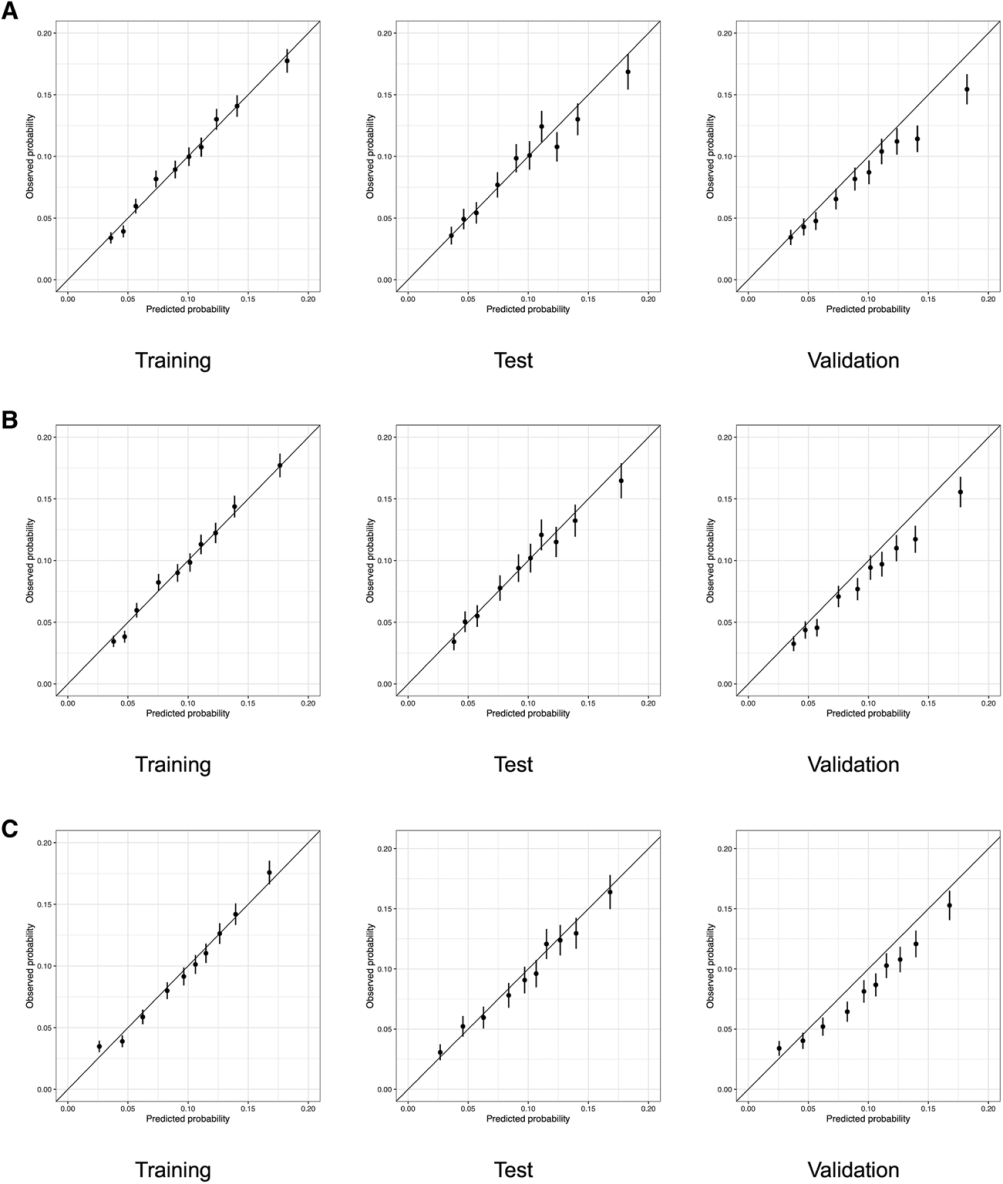
Calibration plots for logistic regression (A), Lasso regression (B), and XGBoost (C) across training, test, and validation cohorts.

Figure 3 displays the calibration plots for logistic regression, Lasso regression, and XGBoost across the training, test, and validation cohorts. Notably, in the validation cohorts, all 3 models tended to overestimate the risk in groups with higher predicted risk levels.

## 4. Discussion

In this study, we developed and validated machine learning models to predict anastomotic leaks using data from the NCD. This represents the first attempt to create a machine learning prediction model leveraging NCD data. The predictive performances of both Lasso regression and XGBoost, with AUROCs of 0.63 each, were found to be suboptimal and not statistically significantly different from that of logistic regression, which had an AUROC of 0.63.

The most important difference between traditional models and machine learning models is their approach to model generation. While traditional models take a model-driven approach, machine learning models take a data-driven approach.^[19]^ By adopting a data-driven approach, machine learning models typically excel in handling interactions and nonlinear relationships among variables, offering superior predictive capabilities compared to traditional models However, this study did not observe any enhancement in prediction accuracy with the machine learning methodology. The suboptimal predictive performance may be attributed to several factors. A recent study employing machine learning algorithm (XGBoost) to National Surgical Quality Improvement Program database, NSQIP risk calculator, showed better discrimination and calibration in the XGBoost model compared to the logistic regression model for predicting various postoperative complication.^[20]^ The difference from our study was that, while the previous study predicted operation-nonspecific complication, our study estimated operation-specific complication. The outcomes in the previous study did not include colectomy specific complication, such as anastomotic leaks. In general, XGBoost algorithm is advantageous when applied to high-dimensional and heterogeneous datasets.

Another possible explanation for limited ability of our mode is that the NCD might lack critical variables for predicting anastomotic leaks. A prior study by the Japanese Society for Colon and Rectal Cancer, which developed a nomogram for anastomotic leak prediction, achieved an AUROC of 0.72.^[5]^ This nomogram incorporated tumor diameter and location relative to the anal verge – factors not considered in our study – suggesting their significant predictive value.

The absence of improved prediction with machine learning in this study suggests a potential lack of complex interactions among the included variables. Another consideration is the possible greater relevance of intraoperative data, such as operative duration, blood loss, and surgeon experience, over preoperative information for predicting anastomotic leaks. While some studies have reported high predictive accuracies using intraoperative details,^[21,22]^ these involved smaller cohorts and specific intraoperative factors. Given the challenges highlighted by previous research and the findings from this study, accurately predicting anastomotic leaks preoperatively remains challenging.^[5,6]^

Lasso regression was not effective in significantly reducing the number of variables. In terms of variable importance, factors such as advanced age, male gender, preoperative sepsis, significant weight loss, hemodialysis, reduced serum albumin levels, and preoperative radiation emerged as key predictors. These findings align with clinical expectations and corroborate previous research utilizing NCD data.^[6]^ Intriguingly, the absence of PT-INR values and a platelet count exceeding 350 × 10^3^/µL also surfaced as significant predictors, suggesting they might act as proxies for other crucial but unmeasured predictors. For this study, we relied on preoperative data from the NCD, as intraoperative and postoperative details would not be applicable for patient consultations prior to surgery.

A prior study that developed a prediction model with NCD data, employing logistic regression and stepwise selection of predictors, achieved an AUROC of 0.64, which aligns closely with the outcomes of our research. This earlier study utilized data from 2011 to 2013, predating the timeframe of our analysis (2015–2021). A potential reason for our prediction model overestimating the risk in the validation cohort could be the relatively high incidence of anastomotic leaks noted in the earlier data set, with rates of 9.73% observed from 2015 to 2021, compared to 9.24% from 2011 to 2013.

Recent advancements in surgical technology are anticipated to reduce the incidence of anastomotic leaks. Robotic surgery – by offering improved dexterity, enhanced visualization, and superior access to the deep pelvis – may be particularly beneficial in high-risk patients, such as those with obesity or a narrow pelvic anatomy.^[23,24]^ However, recent randomized clinical trials and meta-analyses have not demonstrated a significant difference in outcomes between robotic and conventional laparoscopic surgery for rectal cancer.^[25–28]^ Another notable advancement is the intraoperative assessment of perfusion using indocyanine green, which has been associated with a decreased rate of anastomotic leaks.^[29,30]^ Meanwhile, as the use of preoperative therapies – including neoadjuvant chemotherapy and chemoradiotherapy, particularly total neoadjuvant therapy – continues to expand, the preoperative prediction of anastomotic leaks is becoming increasingly complex.

This study presents several limitations that merit acknowledgment. Firstly, essential variables such as tumor location and size were not included in the database, potentially affecting the predictive accuracy. Secondly, our analysis did not distinguish between anastomotic leaks occurring with or without ileostomy. Prior meta-analyses have demonstrated that diverting ileostomy can mitigate the severity of anastomotic leaks and the necessity for reoperation. Regrettably, our dataset lacked information on the frequency of diverting ileostomy among patients. It’s conceivable that the validation cohort had a higher incidence of ileostomy, which might account for the overestimation of risk observed. Furthermore, the study did not classify the severity of anastomotic leaks, for example, using the Clavien–Dindo classification. Thirdly, while Lasso regression helped identify significant predictors that are typically interpretable as risk factors for anastomotic leaks, our study did not establish a causal relationship between these variables and the outcome.

## 5. Conclusion

The machine learning prediction models, including XGBoost and Lasso regression, aimed at forecasting anastomotic leaks using routinely entered preoperative information in the NCD, demonstrated suboptimal predictive performance. This highlights the challenge in accurately predicting anastomotic leaks preoperatively, a fact that could be communicated to patients.

## Author contributions

**Conceptualization:** Takashi Sakamoto, Hideki Endo, Hiroyuki Yamamoto, Yosuke Fukunaga.

**Data curation:** Takashi Sakamoto, Hideki Endo, Hiroyuki Yamamoto.

**Formal analysis:** Hideki Endo.

**Investigation:** Takashi Sakamoto.

**Methodology:** Takashi Sakamoto, Hideki Endo, Hiroyuki Yamamoto.

**Project administration:** Takashi Sakamoto.

**Writing – original draft:** Takashi Sakamoto.

**Writing – review & editing:** Takashi Sakamoto, Hideki Endo, Hiroyuki Yamamoto, Takashi Akiyoshi, Ken Shirabe, Hideki Ueno, Hiroshi Hasegawa, Takeshi Naitoh, Yosuke Fukunaga.
